# An episomal CRISPR/Cas9 system to derive vector-free gene modified mammalian cells

**DOI:** 10.1007/s13238-016-0299-9

**Published:** 2016-07-29

**Authors:** Linlin Li, Fei Gao, Sen Wu

**Affiliations:** State Key Laboratory of Agrobiotechnology, College of Biological Sciences, China Agricultural University, Beijing, 100193 China

**Dear Editor,**

CRISPR and CRISPR-associated (Cas) proteins play their adaptive immunity role in degrading foreign nucleic acids in both bacteria and archaea. CRISPR/Cas has proved efficient in modifying mammalian genomes (Cong et al., 2013; Mali et al., 2013) and various delivery methods for Cas9:gRNA complex have been established. Among different delivery means, *in vitro* transcribed Cas9 mRNA/gRNA, purified Cas9 protein, and adeno-associated virus (AAV) driven seem promising toward clinical applications (Kouranova et al., 2016; Lin et al., 2014; Yin et al., 2016). Still, all the fore-mentioned methods for delivering CRISPR/Cas9 have certain shortcomings. *In vitro* transcription and protein purification are complicated and inconvenient for use, while AAV mediated system suffers from the side effects of immune response and insertional mutagenesis (Donsante et al., 2007; Li et al., 2011).

Episomal vectors based on oriP-*EBNA1* have been shown as an effective method to derive vector-free cells in reprogramming studies (Okita et al., 2011; Yu et al., 2009). Without selection pressure, EBV vectors are gradually lost in each generation due to their defects in synthesizing and partitioning thus resulting in transgene-free daughter cells (Nanbo et al., 2007). We thus set out to test if Cas9 protein and gRNA can be effectively delivered as episomes to generate vector-free mutations in mammalian cells. Here we report efficient human genome editing with CRISPR/Cas9 technology in the form of oriP-*EBNA1*-based episomes. To obtain oriP*-EBNA1*-based CRISPR/Cas9 vectors, we first assembled CMV-FLAG-hSpCas9-2A-GFP and U6-gRNA and oriP*-EBNA1* fragments together. To further enrich successfully transfected cells, we added SV40-puro to form pCRISPR-S12 (Fig. 1), which hereafter is referred to as oriP*-EBNA1*-based CRISPR/Cas9 vector or system.

**Figure 1:**
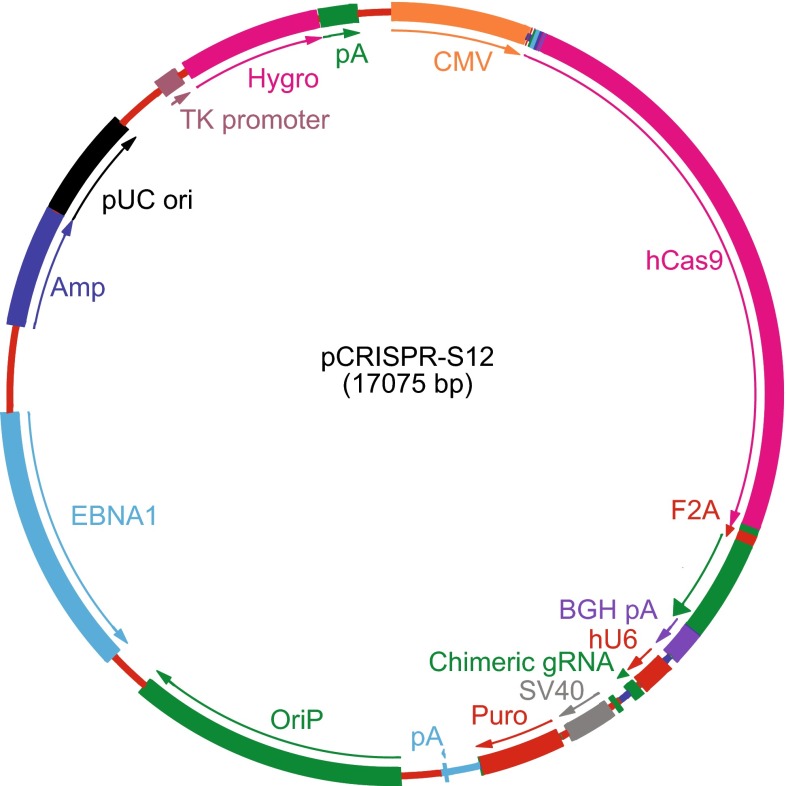
Circular map of oriP*-EBNA1*-based CRISPR/Cas9 vector. CMV, cytomegalovirus promoter; 3× FLAG, FLAG epitope tag; hCas9, humanized *Streptococcus* pyogenes Cas9; F2A, 2A regions of foot-and-mouth disease virus; GFP, green fluorescent protein gene; BGH pA, bovine growth hormone polyA signal; U6, human U6 promoter; chimeric gRNA, synthetic gRNA scaffold; oriP, Epstein-Bar virus (EBV) latent origin of replication; *EBNA1*, EBV nuclear antigen-1, replication transactivator of EBV; SV40, SV40 promoter; Puro, puromycin resistant gene; Amp, ampicillin resistant gene; pUC ori, replication origin; TK promoter, thymidylate kinase promoter; Hygro, hygromycin resistant gene; pA, polyA signal.

To test the oriP*-EBNA1*-based CRISPR/Cas9 system, we first examined its efficiency for disrupting single-copy tdTomato expression driven by CAG promoter in mouse induced pluripotent stem cells (miPSCs). We designed crtdTomato to target tdTomato coding sequence with the expectation of destructing an existing Sau3AI restriction enzyme site (Fig. 2A). The tdTomato-tagged-miPSCs were transfected with pCRISPR-S12-crtdTomato and manipulated according to the flowchart (Fig. 2B). We observed disappearance of tdToamto fluorescence in some of the miPSCs clones under a fluorescent microscope (Fig. 2C). Further, flow-activated cell sorting (FACS) showed a reduction of 26% tdTomato-labeled cells in crtdTomato treated group when compared with non-transfected group five days post transfection (Fig. 2D). These results suggested that our system works in inactivating reporter genes in miPSCs.

**Figure 2:**
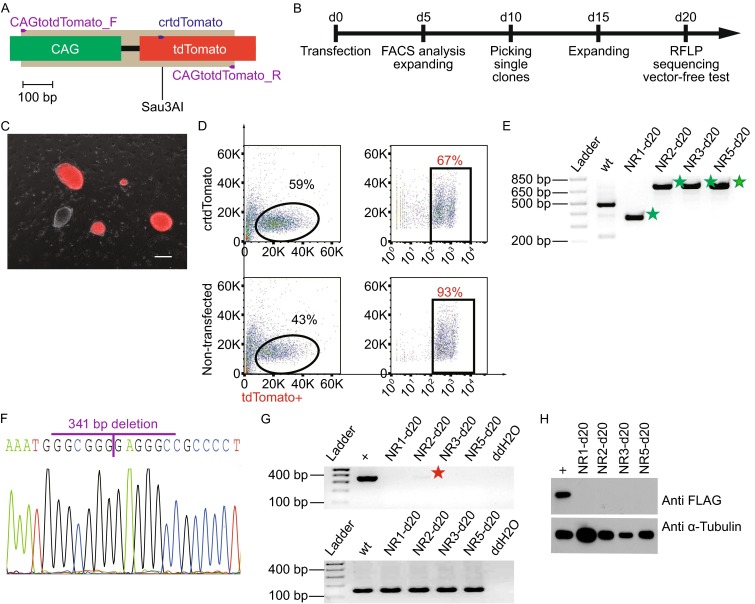
oriP*-EBNA1*-based CRISPR/Cas9 disrupted fluorescent expression in miPSCs. (A) Schematic of crtdTomato targeting site (blue arrow), primers used (purple arrows) for amplifying genomic sequences flanking target sites (light gray boxes), CAG promoter (green box) drove the expression of tdTomato (red box), and Sau3AI restriction sites were used for RFLP assay. (B) Schematic of the experimental procedure. (C) Disappearance of red fluorescence in partial tdTomato-labelled-miPSCs clones. Scale bar, 200 μm. (D) FACS analysis of crtdTomato transfected and non-transfected miPSCs. The upper two panels are crtdTomato transfected group (tdTomato + with a portion of 67%) and the lower two panels are non-transfected group (tdTomato+ with a portion of 93%). (E) RFLP of non-red (NR) clones by Sau3AI. The green pentagrams indicated the uncut PCR amplicons by Sau3AI. Ladder, 1 kb plus ladder in all figures in this study. (F) Sanger sequencing confirmed deletion of 341 bp of NR1-d20 clone. The purple line between G/G showed the deleted site. (G) PCR confirmed the removal of *EBNA1* fragment in clone NR1-d20, NR2-d20, NR5-d20 but not in NR3-d20. pCRISPR-S12 DNA was used as positive control (“+” in all figures in this study). “wt” stands for wild type mouse genomic DNA. Red pentagram shows visible EBNA1 residue in NR3-d20. (H) Western blot showed no hSpCas9 expression in clone NR1-d20, NR2- d20, NR3-d20, and NR5- d20. HeLa cells expressing FLAG-hSpCas9-2A-GFP stably was used as positive control (“+” in all figures in this study). The expected protein band is about 190 kDa.

Next, we further detected the targeting efficiency of our system in 6 non-red (NR) single clones. We were able to PCR amplify the target regions in 4 out of the 6 NR clones. Clones NR4 and NR6 were not analyzed further since PCR failed to produce products (Fig. S1). PCR products of the remaining 4 clones were confirmed as expected mutants by restriction fragment length polymorphism (RFLP) assays (Fig. 2E and 2F). Among these, clone NR1 showed a truncated size (Fig. S1), which was further confirmed by Sanger sequencing as a 341 nucleotides deletion (Fig. 2F). Above data showed that our oriP*-EBNA1*-based CRISPR/Cas9 system could mediate efficient gene disruption.

Finally, we examined whether these four successfully engineered miPSCs (NR1, NR2, NR3, NR5) were free of targeting vector. PCR analysis showed that 3 clones were free of vectors, but clone NR2-d20 still retained residual amount of vectors (Fig. 2G). However, Western blot of FLAG-hSpCas9-2A-GFP further verified no continuous expression of hSpCas9 protein of all four clones (Fig. 2H), suggesting that PCR detection was more sensitive in detecting foreign gene expression than Western blot assay. Together, these results demonstrated that our oriP*-EBNA1*-based CRISPR/Cas9 system inactivated reporter gene expression as expected, and more importantly, vector-free engineered cell lines can be readily obtained.

We further attempted to examine whether our system works well in human cells. The basic functions of CRISPR/Cas9 were confirmed by deleting accurately more than 2 kb intervening nucleotides of human chemokine receptor 5 (*CCR5*) as well as editing multiplex genes (human *TET1*, *TET2*, and *TET3*) simultaneously (Figs. S3–S6 and Table S1). Moreover, our oriP*-EBNA1*-based CRISPR/Cas9 system was free of vector in both genetically modified miPSCs and human cells. Together, these results demonstrated the feasibility of manipulating mouse and human genomes with our oriP*-EBNA1*-based CRISPR/Cas9 system.

In the current study, we have verified the functionality of our episomal CRIPSR/Cas9 system first by inactivating visible reporter gene, and further by specifically deleting a single genomic region and editing multiplex genes in a transgene-free manner.

One potential risk for CRISPR/Cas9 in genome editing is its uncertain off-target effects which could confound genome-editing-based therapies. In present study, we detected certain frequencies (none for cr*TETs* and 10% for cr*CCR5*) of off-target events by T7EI assay (Fig. S7 and Table S2). In the future, combined with other progresses, such as mutant Cas9 or new homologues and optimized gRNA design, episomal CRISPR/Cas9 could further ameliorate, if not eliminate, the off-target influences, thereby broadening its application.

## Electronic supplementary material

Below is the link to the electronic supplementary material.

Supplementary material 1 (PDF 10699 kb)
