# Supplementary material for: An episomal CRISPR/Cas9 system to derive vector-free gene modified mammalian cells
Source: Protein Cell. 2016 Jul 29;7(9):689–91. doi: 10.1007/s13238-016-0299-9 (PMC5003789; doi:10.1007/s13238-016-0299-9)
Supplement: Supplementary file 1 — Supplementary material 1 (PDF 10699 kb) [file 13238_2016_299_MOESM1_ESM.pdf]

Materials and Methods

Construction of the final vectors

Fragment of oriP-*EBNA1* was cloned from pCEP4 (Thermo).

Various crRNAs were designed by online CRISPR design tool (http://tools.genome-engineering.org), then oligos were ordered (see Table S3) and annealed in a thermalcycler, the annealed crRNAs were cloned into pX330BB (pX330-U6-Chimeric_BB-CBh-hSpCas9 backbone digested by BbsI) to form pX330-gRNA firstly. Primers containing Not I and BamH I restriction enzyme sites (see Table S3, Primer-1) were used to amplify fragments from pX330-gRNA. The fragments were purified with the QIAprep spin miniprep kit (Qiagen) as the manufacturer's demand. Then purified PCR fragments were digested by Not I and BamH I and purified by QIAquick gel extraction kit (Qiagen), the former purified fragments were cloned into pCRISPR-S12BB (pCRISPR-S12 digested by NotI and BamHI) to form the final pCRISPR-S12 which expresses hSpCas9 and specific single guide RNAs (gRNAs) plus oriP-EBNA1 elements for different target sites. All pCRISPR-S12 based vectors used in this study will be deposited to Addgene for wide distribution.

Cell culture

miPS cell line stably integrated piggyBac-tdTomato was cultured on irradiated beta2 feeder cells in 2i (CHIR99021 and PD0325901, Selleck) plus mouse LIF. The cells were dissociated by TryplE Express (Thermo) and expanded every 5 days.

HeLa cells (CLS, Cell Lines Service) were maintained in Dulbecco's modified Eagle medium (DMEM, Invitrogen) supplemented with 10% (vol/vol) fetal bovine serum (FBS), 1% penicillin–streptomycin (Gibco) and 1% non-essential amino acids (Invitrogen). The cells were dissociated by TryplE Express (Thermo) and expanded every 3 days.

The cells were incubated in a humidified 37°C, 5% CO_2 incubator. The culture media were replaced every day.

Transfection

pCRISPR-S12-crtdTomato (4 µg) was electroplated into ~10^5 tdTomato-labeled mouse induced pluripotent stem cells with Nucleofector™ 2b Device (Lonza) using program A-030.

Appropriate amount of pCRISPR-S12-crRNA vector (4 µg for single vector, 2 µg for each of the two vectors, 1.33 µg for each of the three vectors) was electroplated into ~10^6 HeLa cells with Nucleofector™ 2b Device (Lonza) with program I-013.

FACS analysis

Five days post transfection by pCRISPR-S12-crtdTomato, percentage of tdTomato+ cells were analysed by a BD FACSCalibur™ system and CellQuest™ software (Becton Dickinson), non-transfected miPSCs were used as the control group.

Cell lysis and genomic PCR

Cultured cells were treated with 500 µl ES lysis buffer (100 mM Tris-HCl pH8.5, 200 mM NaCl, 5 mM EDTA, 0.2% SDS) and 100 ng/ml proteinase K for 3 to 4 hours at 37°C. The genomic DNA were extracted as standard protocol. After an initial treatment in thermal cycler (99°C for 10 minutes), genomic PCR was carried out using primers listed in Table S3 with Herculase II Fusion Polymerase (Stratagene) as the manufacturer's instructions.

Detection of indel mutation

Indel rates of crRNAs were analyzed by Surveyor Mutation Detection Kit (IDT). The indel rates were calculated according to the following formula: Indel rate (%) = $(1 - \sqrt{(1 - r)}) \times 100$, where $r = (a+b)/(a+b+c)$. In this formula, "a" and "b" represent the gray value of the cleaved bands while "c" represents the gray value of the un-cleaved bands.

Sequencing of the genomic mutations was done after cloning the corresponding amplicons into pMD19-T vector (Takara). The digested fragments by RFLP were resolved on a 2% agarose gels while the digested fragments of Surveyor assay were resolved on polyacrylamide gel.

Off-target analysis

We chose top 3 off target sites for each crRNA in all samples for further analysis (all primers were listed in Table S3). Genomic sequences flanking potential off target sites were amplified and purified with QIAprep spin miniprep kit (Qiagen) following the manufacturer's strctions. Then, we performed T7EI assay according to the manufacturer's demand and the digested fragments were dissolved.

Detection of *EBNA1* remnant

The primers (Primer-2) we used for the examination of *EBNA1* element of our vector were listed in Table S3. The PCR were carried out with Gotaq Flexi DNA Polymerase (Promega) in accordance with the manufacturer's instructions.

Western blot

After washing with PBS, the cells of expanded cells were washed with PBS, then each well was treated with appropriate volume of RIPA cell lysis buffer (PMSF was added). Total protein of each sample was measured by BCA protein Kit. Protein samples were boiled at 99°C for 10 min, then were separated by 10% SDS-PAGE and transferred to Immobilon-P transfer membrane (Millipore). The membrane was blocked by 5% non-fat milk at room temperature for 1h. Then specific antibodies against Tubulin (1:4,000, mouse monoclonal, Beyotime) and FLAG (1:2,000, monoclonal, Beyotime) were incubated with membrane at 4°C overnight. Next the membrane was washed for six times and incubated with HRP labeled goat-anti-mouse IgG (1:5,000, Beyotime) at room temperature for 2h. Finally the membranes were washed six times again and incubated with SuperSignal West Dura Extended Duration Substrate (Thermo) and exposed to X-ray film (Fujifilm) for appropriate time.

Supplementary Figure Legends

Figure S1 PCR of crtdTomato flanking region and internal mouse *GAPDH*

A) HerII polymerase amplified crtdTomato flanking region of NR1 to NR6 clones. Three repeats for each sample.

B) Internal mouse *GAPDH* of wt and NR1 to NR6. wt, short for wild type mouse genomic DNA.

Figure S2 Sequence of NR2, NR3 and NR5 mutant clones

A) Sequencing of NR2 mutant clone showed AT>GG alteration. GATC was original sequence of restriction enzyme site for Sau3AI in Figure S2.

B) Sequencing of NR3 mutant clone showed TCGAGA deletion.

C) Sequencing of NR5 mutant clone showed CGAGTTCGAGAT deletion.

Figure S3 oriP-*EBNA1*-based CRISPR/Cas9 mediated accurate deletion in HeLa cells without residual foreign DNA and hSpCas9 protein

A) Schematic of oriP-*EBNA1*-based CRISPR/Cas9 targeting sites at the *CCR5* locus (black rectangular stands for exons in all figures in this study). The top and bottom panels used 10 mm to represent 100 nucleotides but not in the middle.

B) crE2-1 and crE2-2 deleted 205 nucleotides of *CCR5* at the second exon. Red dCCR5 represented the truncated bands surrounded by red dotted line (in this figure).

C) Different crRNA pairs mediated accurate deletion of 2230 bp (crE1-1 plus crE2-1), 2435 bp (crE1-1 plus crE2-2), 2242 bp (crE1-2 plus crE2-1), 2447 bp (crE1-2 plus crE2-2).

D) Schematic procedure of deriving single clones transfected by crE1-1 plus crE2-1.

E) PCR confirmation of truncated clones by crE1-1 plus crE2-1. The 537 bp band was presented in CCR5-16 and CCR5-19 at day 12 post transfection.

F) PCR confirmation of removal of *EBNA1* fragment in clone CCR5-16, CCR5-19 at day 12 and day 15 post transfection respectively..

G) Western Blot detected no continuous hSpCas9 expression in clone CCR5-16 at day 12 and CCR5-19 at day 15 post transfection.

Figure S4 Sanger sequencing confirmed accurate deletion of *CCR5* by crRNA pairs

A) Accurate deletion of 205 bp fragment by crE2-1 plus crE2-2 were confirmed by Sanger sequencing. Predicted cut sites were guided by red arrows, genomic sequences were in black and PAMs were in light blue (in Figure S4).

B) Accurate deletion of >2 kb fragment by crRNA pairs were confirmed by Sanger sequencing.

Figure S5 oriP-*EBNA1*-based CRISPR/Cas9 mediated multiplex knockout in HeLa cells without foreign vector remnant

A) Schematic of oriP-*EBNA1*-based CRISPR/Cas9 targeting sites at the *TET1*, *TET2* and *TET3* loci. StyI and PstI were restriction sites used for RFLP assay of *TET1* and *TET2* respectively.

B) Surveyor assay for oriP-*EBNA1*-based CRISPR/Cas9 mediated cleavage in HeLa cells using single crRNA targeting *TET1* (cr*TET1*), *TET2* (cr*TET2*) and *TET3* (cr*TET3*). The red pentagrams represents the expected mutant bands used to calculate indel rates (blue in this figure).

C) RFLP assay for oriP-*EBNA1*-based CRISPR/Cas9 mediated cleavage in HeLa cells using single gRNA targeting *TET1* (cr*TET1*), *TET2* (cr*TET2*) and three crRNAs targeting three loci (crmix). The green pentagrams represents the expected mutant band used to calculate indel rates.

D) Schematic procedure for deriving 3-KO clones in HeLa cells.

E) Sanger sequencing of HeLa cells transfected by three crRNAs confirmed indels for different target sites. PAMs were indicated in light blue and mutant sites were in red.

F) Percentage of 1-KO (one gene knockout), 2-KO (two genes knockout) and 3-KO (three genes knockout) clones in all mutant HeLa cell lines.

G) Removal of *EBNA1* fragment examination in five clones at day 15 and day 18 by PCR detection.

H) Western Blot confirmation of no hSpCas9 expression in clone TETs-1 at day 15 and TETs-6 at day 18 respectively.

Figure S6 Surveyor assay for crmix-transfected compound and detection of vector-free for 3-KO cells

A) Surveyor assay for oriP-*EBNA1*-based CRISPR/Cas9 mediated cleavage in HeLa cells using all crRNAs targeting three loci (crmix).

B) PCR detection of *EBNA1* residue in 3-KO cell lines at day 12.

C) Western Blot detection of hSpCas9 expression in 3-KO clones at day 12.

Figure S7 T7EI assay of TETs and CCR5 top3 off-target sites

T7EI assay of crTETs and crCCR5 potential off-target sites showed no off-target for cr*TET*s and 10% off-target for cr*CCR5*. ctr was short for control or wild type group,

OT was short for predicted off-target sites, red pentagram showed detected off-target sites.

Figure S1 PCR of crtdTomato flanking region and internal mouse GAPDH

A

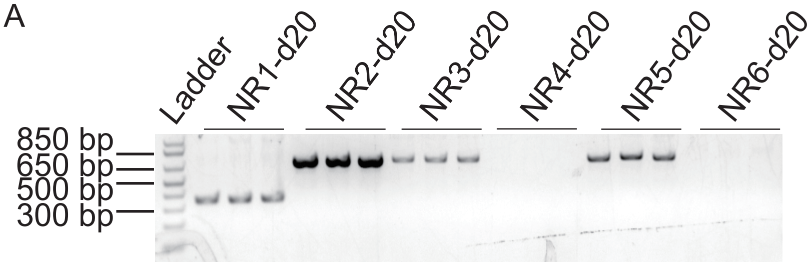

B

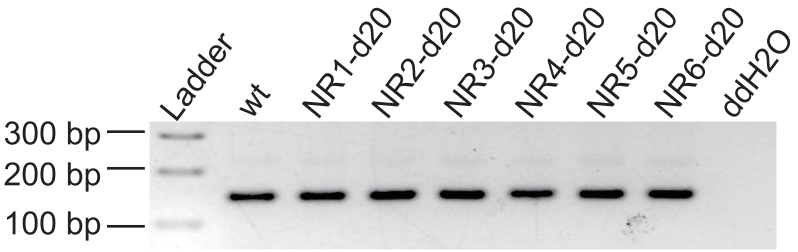

Figure S2 Sequence of NR2, NR3 and NR5 mutant clones

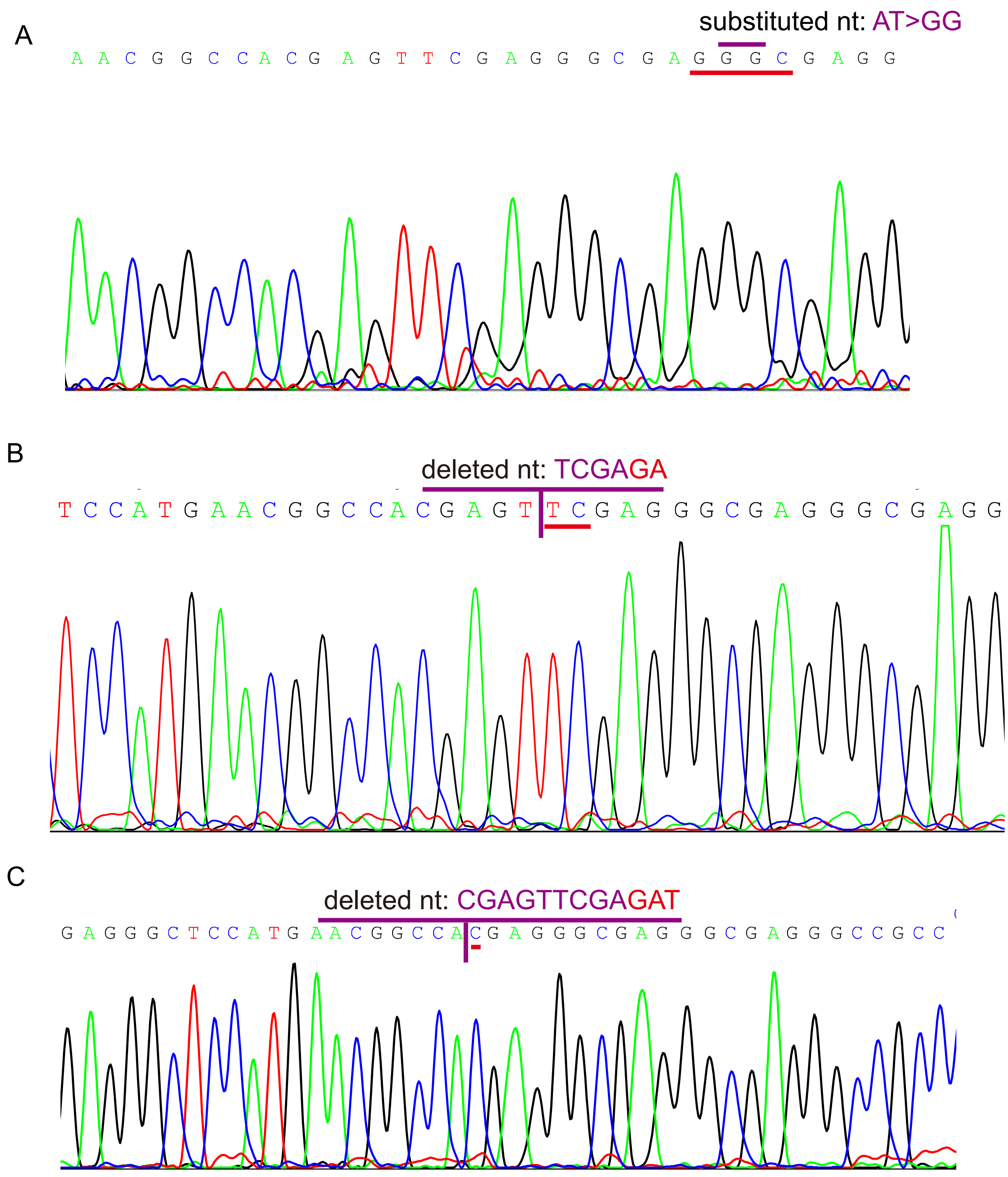

Figure S3 oriP-EBNA1-based CRISPR/Cas9 mediated accurate deletion in HeLa cells without residual foreign DNA and hSpCas9 protein

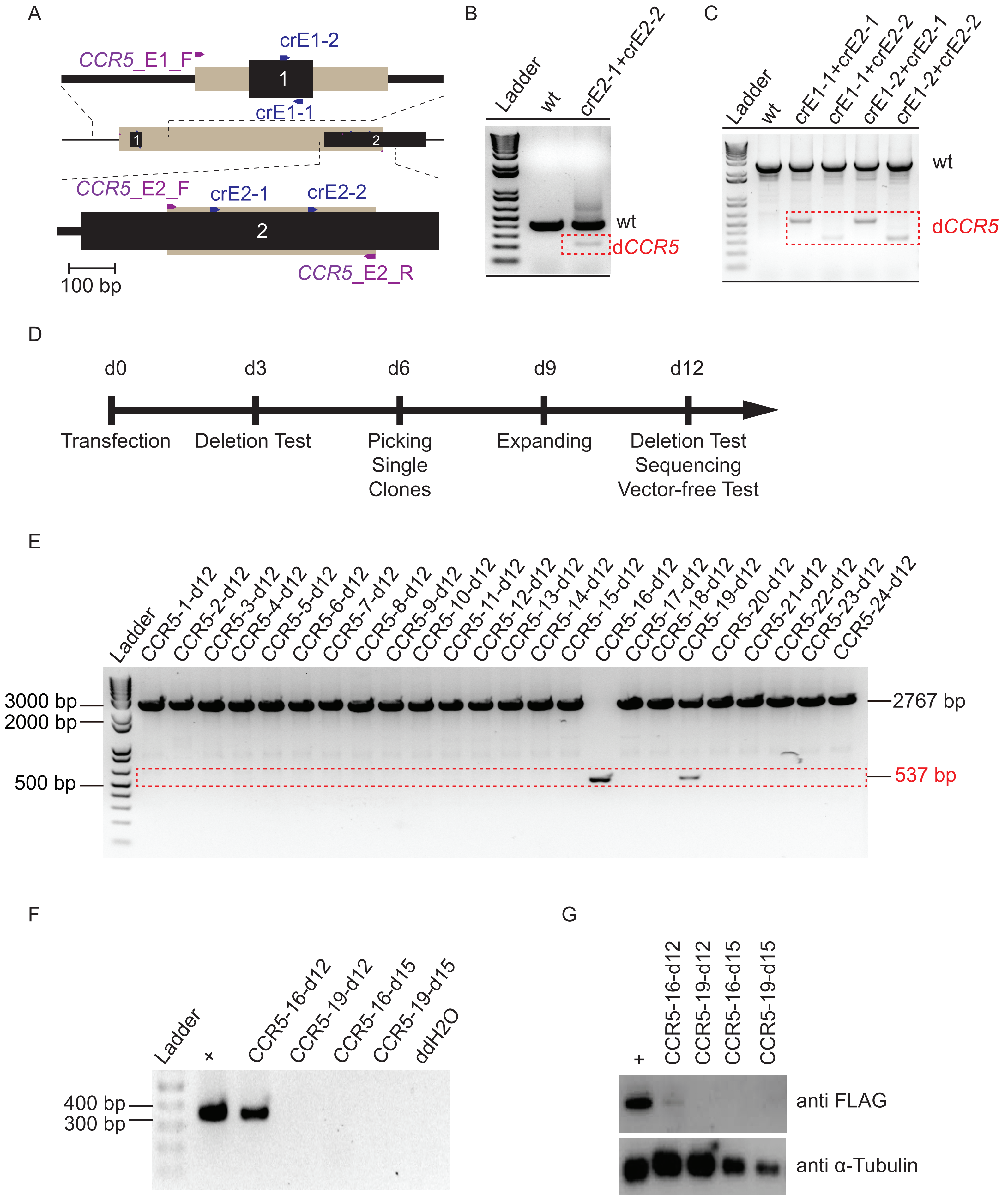

Figure S4 Sanger sequencing confirmed accurate deletion of CCR5 by crRNA pairs

A

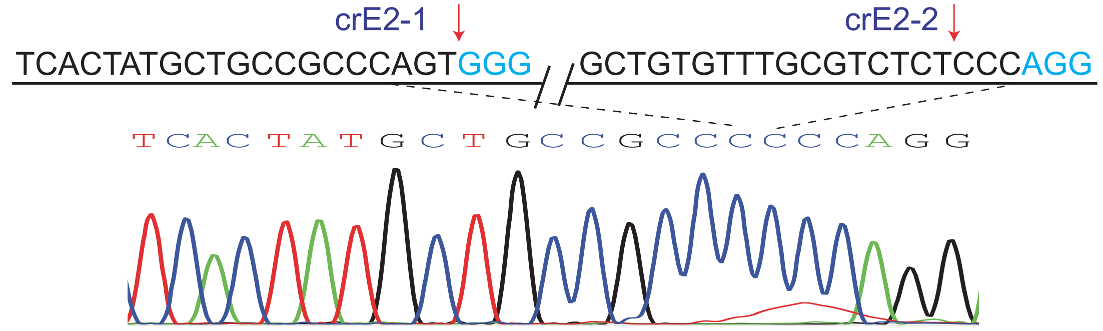

B

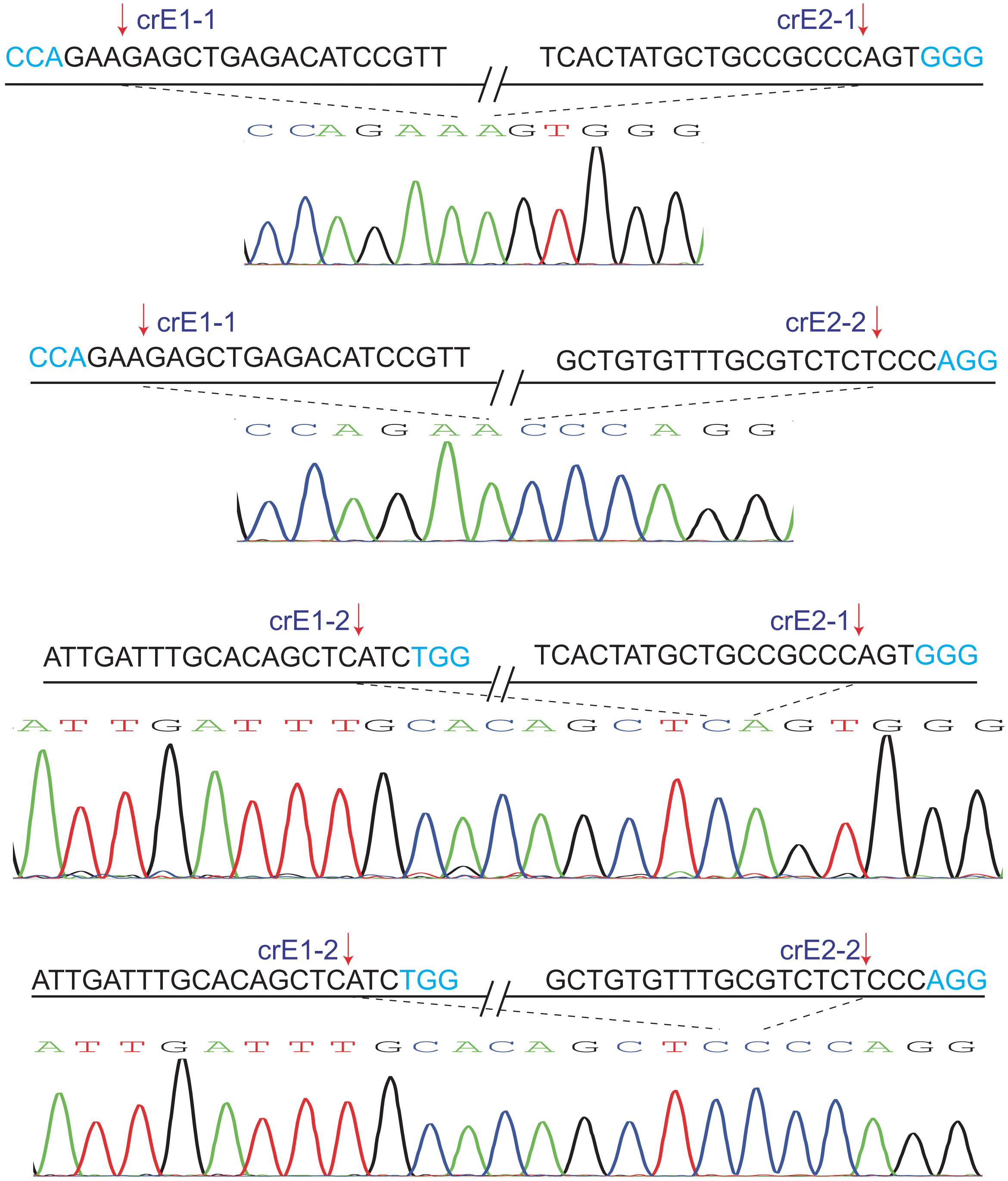

Figure S5 oriP-EBNA1-based CRISPR/Cas9 mediated multiplex knockout in HeLa cells without foreign vector remnant

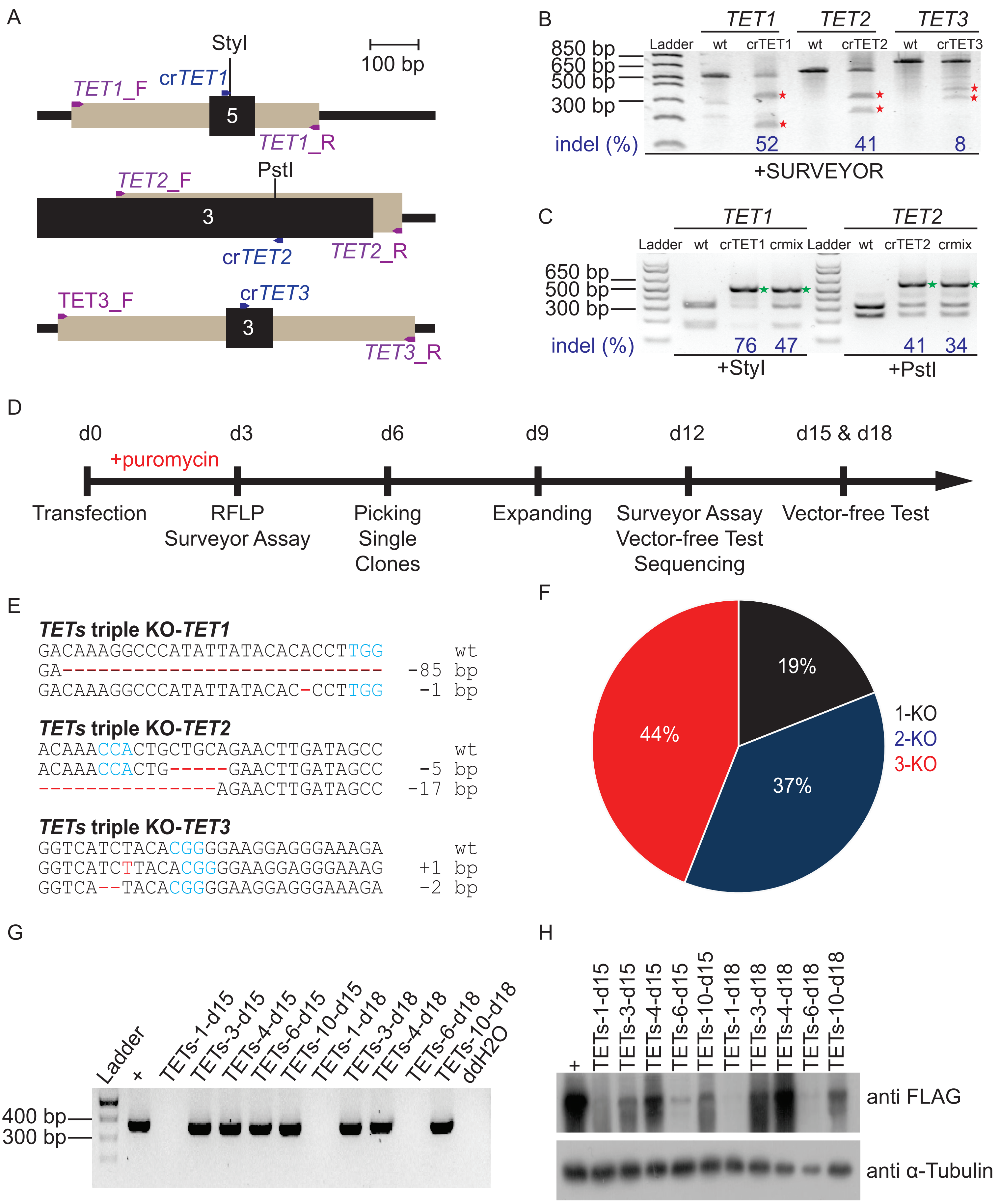

Figure S6 Surveyor assay for crmix-transfected compound and detection of vector-free for 3-KO cells

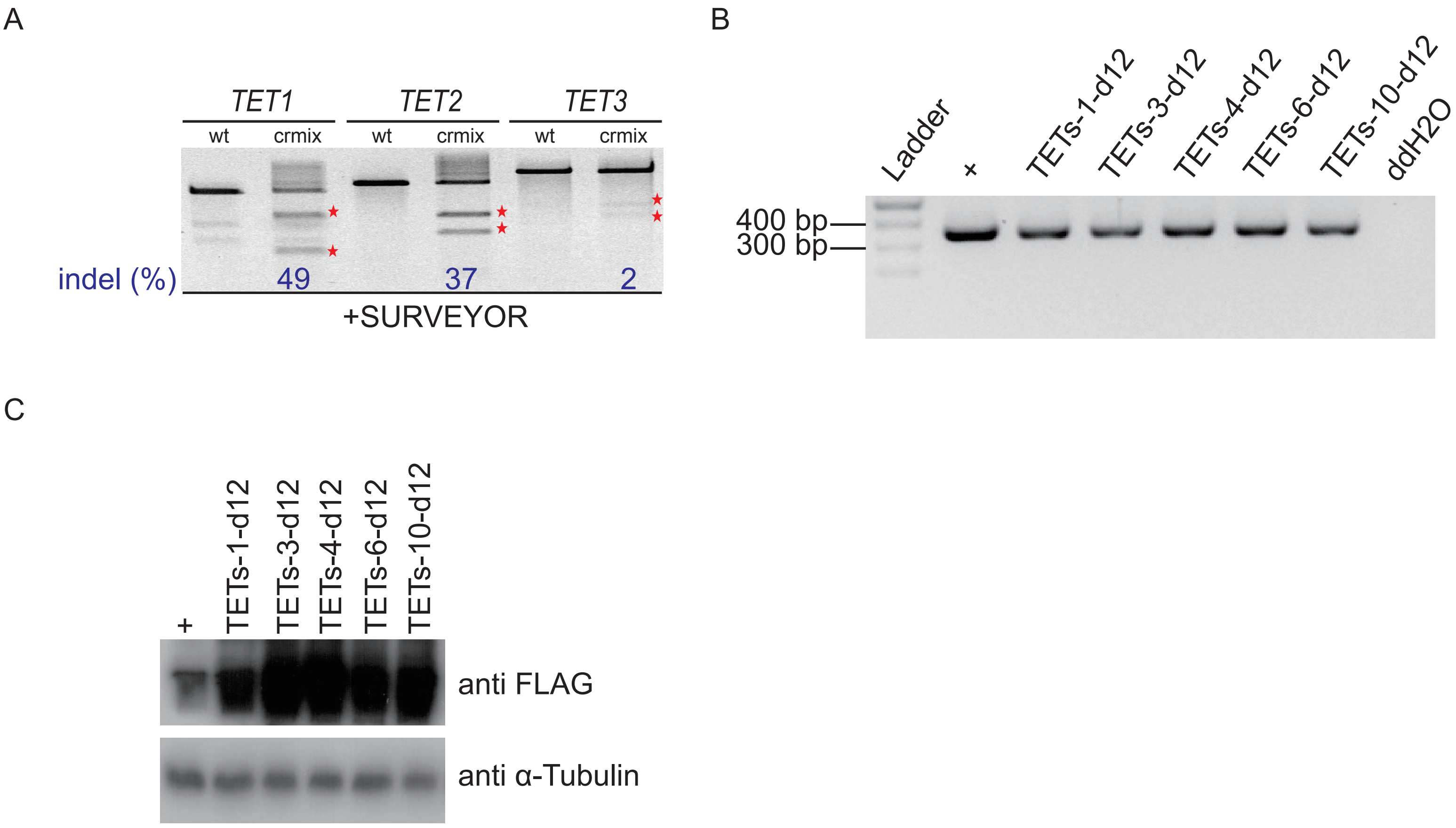

Figure S7 T7EI assay of TETs and CCR5 top3 off-target sites

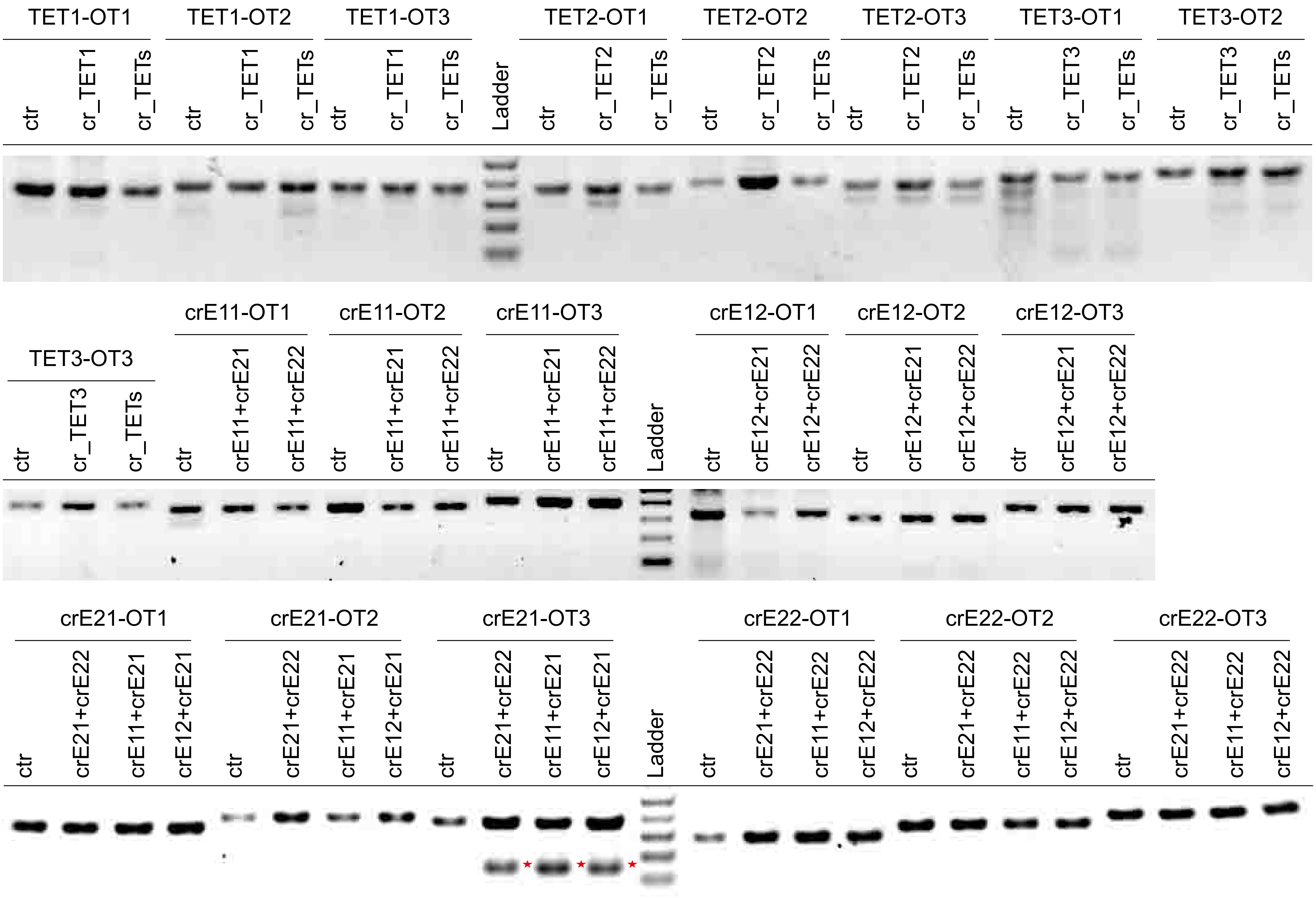

Supplementary Tables

Table S1 Detection of transfected HeLa cell lines (cr*TET1*, cr*TET2* and cr*TET3*) for knockout situations at day 15 post transfection

Clone ID (d15)	*TET1* KO	*TET2* KO	*TET3* KO
TETs-1	Y	Y	Y
TETs-2	Y	N	Y
TETs-3	Y	Y	Y
TETs-4	Y	Y	Y
TETs-5	Y	Y	N
TETs-6	Y	Y	Y
TETs-7	Y	Y	N
TETs-8	Y	N	Y
TETs-9	Y	Y	N
TETs-10	Y	Y	Y
TETs-11	Y	Y	N
TETs-12	Y	Y	Y
TETs-13	Y	Y	Y
TETs-14	Y	Y	Y
TETs-15	Y	Y	Y
TETs-16	Y	Y	N
TETs-17	Y	N	N
TETs-18	Y	N	Y
TETs-19	Y	Y	Y
TETs-20	Y	Y	N
TETs-21	N	Y	N
TETs-22	Y	Y	N
TETs-23	Y	Y	Y
TETs-24	N	Y	Y
TETs-25	N	Y	N
TETs-26	Y	N	N
TETs-27	Y	Y	Y

Note: Y for detected knockout, N for not detected knockout.

Table S2 Off target by T7EI assay for cr*CCR5* and cr*TETs* (*TET1*, *TET2* and *TET3*)

	Off target site	Total sites tested	incidence
CCR5	3	30	10%
TETs	0	18	0

Table S3 List of oligos

Oligo ID	Oligo Sequence
CAGtotdTomato-Fn	ATTGCCTTTTATGGTAATC
CAGtotdTomato-Rn	GCCGTCCTCGAAGTTCATC
crtdTomato-S	CACC GGCCACGAGTTCGAGATCGA
crtdTomato-A	AAAC TCGATCTCGAACTCGTGGCC
	CAGtotdTomato_F/CAGtotdTomato_R=695 bp
CCR5_E1_F	CCAGTGAGAAAAGCCCGTAA
CCR5_E2_F	CTGCAAAAGGCTGAAGAGCA
CCR5_E2_R	CCCCAAGATGACTATCTTTAATGTC
crE1-1-S	CACC gAACGGATGTCTCAGCTCTTC
crE1-1-A	AAAC GAAGAGCTGAGACATCCGTTc
crE1-2-S	CACC gATTGATTTGCACAGCTCATC
crE1-2-A	AAAC GATGAGCTGTGCAAATCAATc
crE2-1-S	CACC gTCACTATGCTGCCGCCCAGT
crE2-1-A	AAAC ACTGGGCGGCAGCATAGTGAc
crE2-2-S	CACC GCTGTGTTTGCGTCTCTCCC
crE2-2-A	AAAC GGGAGAGACGCAAACACAGC
	CCR5_E1_F/CCR5_E2_R=2767 bp
	CCR5_E2_F/CCR5_E2_R=436 bp
TET1_F	GGAGATAGGAGTATAAATATGACCC
TET1_R	GCCCTAAGAAACATCCAACTC
crTET1-S	CACC GGCCCATATTATACACACCT
crTET1-A	AAAC AGGTGTGTATAATATGGGCC
	TET1_F/TET1_R=498 bp
TET2_F	ACACAGCAACCCCAAACTG
TET2_R	TTGCTAATTCTGGATAAACGC
crTET2-S	CACC GCTATCAAGTTCTGCAGCAG
crTET2-A	AAAC CTGCTGCAGAACTTGATAGC
	TET2_F/TET2_R=568 bp
TET3_F	ACAAATGCCCATGTTTAGCC
TET3_R	CCTTGGAACTGTGTTAGTCTTCA
crTET3-S	CACC GATCGAGAAGGTCATCTACA
crTET3-A	AAAC TGTAGATGACCTTCTCGATC
	TET3_F/TET3_R=707 bp
Primer-1-F	accggtccctGCGGCCGCAAGCTTtttcccatgattccttcatatttg
Primer-1-R	ccttgattagGGCGCGCCGGATCCacgcgctaaaaacggactagcctt
	Primer-1-F/Primer-1-R=442 bp
Primer-2-F	GGAAACCAGGGAGGCAAATCTACT
Primer-2-R	TCACGTAGAAAGGACTACCGACGA
	Primer-2-F/Primer-2-R=357 bp
mGAPDH-F	AAGGTCATCCCAGAGCTG
mGAPDH-R	CCTGCTTCACCACCTTCTTG
	mGAPDH-F/R=139 bp
crTET1-OT1-F	tccccatgagattattggatt
crTET1-OT1-R	ctaatcagtgttattgctcctgtca
	crTET1-OT1-F/R=352 bp
crTET1-OT2-F	ttgagcccaggagttcattc
crTET1-OT2-R	gggaatgcctgttttgtacct

	crTET1-OT2-F/R=367 bp
crTET1-OT3-F	cagttttccacagcaaagca
crTET1-OT3-R	gtggtggcctgtacactgct
	crTET1-OT3-F/R=373 bp
crTET2-OT1-F	ggagaaataaaggagggtgga
crTET2-OT1-R	ttttggtcaggataaacgcc
	crTET2-OT1-F/R=376 bp
crTET2-OT2-F	gaaagagcagacccctcaca
crTET2-OT2-R	actgaagtggtgagcatccc
	crTET2-OT2-F/R=400 bp
crTET2-OT3-F	tcttcttacaggtcccagaacttt
crTET2-OT3-R	tcaggctggtcttgaactcc
	crTET2-OT3-F/R=370 bp
crTET3-OT1-F	cctcttccttgtcctttccc
crTET3-OT1-R	ttcctcctcagacagtgacaaa
	crTET3-OT1-F/R=398 bp
crTET3-OT2-F	gcctcaagtactgaagat
crTET3-OT2-R	ccaaagtataagcatgtgtt
	crTET3-OT2-F/R=418 bp
crTET3-OT3-F	cggaactctaaatatatgagatggc
crTET3-OT3-R	ttggttagtgtgagtccaccc
	crTET3-OT3-F/R=387 bp
crE11-OT1-F	atgtccctgagcatcatttg
crE11-OT1-R	ccaggctaactccacttt
	crE11-OT1-F/R=359 bp
crE11-OT2-F	ctgggaaacttcgtttctgc
crE11-OT2-R	cctgaacagctgttctacaca
	crE11-OT2-F/R=376 bp
crE11-OT3-F	gataggggctgacaggaaca
crE11-OT3-R	tgggctctgttttagttgcc
	crE11-OT3-F/R=397 bp
crE12-OT1-F	gtagaatgcagcttcctggc
crE12-OT1-R	ggcccagatctacactggaa
	crE12-OT1-F/R=326 bp
crE12-OT2-F	tcatgacccatgaccatgac
crE12-OT2-R	tcatgccacattctgtcgat
	crE12-OT2-F/R=305 bp
crE12-OT3-F	gccaggtagagcctcagtcta
crE12-OT3-R	ccgcttgaggcagatatga
	crE12-OT3-F/R=366 bp
crE21-OT1-F	cctcgtcagtaaaatgggga
crE21-OT1-R	catccagccccatcactatt
	crE21-OT1-F/R=334 bp
crE21-OT2-F	caggactgttgagggaggaa
crE21-OT2-R	gagacattgctccaggaacc
	crE21-OT2-F/R=395 bp
crE21-OT3-F	ctctttctccgatctgcctg
crE21-OT3-R	cctcgtcagtaaaatgggga
	crE21-OT3-F/R=376 bp
crE22-OT1-F	agaatcggctgctctgtagc

crE22-OT1-R	cctttggatcctgtctggtg
	crE22-OT1-F/R=292 bp
crE22-OT2-F	cccttctctccattcacagg
crE22-OT2-R	tgctttggcagtttgcacta
	crE22-OT2-F/R=347 bp
crE22-OT3-F	gtagccagcaaggatgaagg
crE22-OT3-R	gtgagagaaggtgccacaca
	crE22-OT3-F/R=389 bp
